# Clinical heterogeneity of ocular Behçet’s syndrome versus intestinal Behçet’s syndrome: a cross-sectional study from Shanghai Behçet’s syndrome database

**DOI:** 10.1186/s13075-022-02782-1

**Published:** 2022-04-29

**Authors:** Cheng-cheng Hou, Dan Luo, Hua-fang Bao, Jing-fen Ye, Hai-fen Ma, Yan Shen, Jun Zou, Jian-long Guan

**Affiliations:** Department of Rheumatology and Immunology, Huadong Hospital affiliated to Fudan University, #221 Yan’an West Road, Shanghai, 200040 People’s Republic of China

**Keywords:** Behçet’s syndrome, Uveitis, Intestinal ulcers

## Abstract

**Background:**

Behçet’s syndrome (BS) is a rare variant vasculitis which can involve the eyes and gastrointestinal systems. However, ocular involvement rarely overlaps with intestinal lesions. This study aimed to compare the clinical characteristics and laboratory parameters of ocular BS and intestinal BS patients in China and analyze the differences between two key phenotypes to verify the heterogeneous conditions in BS patients.

**Methods:**

A retrospective analysis was used to collect the demographic data, clinical characteristics, endoscopic findings, and laboratory parameters from 135 ocular BS and 174 intestinal BS patients. The Mann-Whitney *U* test and Pearson chi-square or continuity correction was used to analyze the differences between two groups.

**Results:**

Among 916 BS patients enrolled in this study, ocular BS and intestinal BS accounted for 14.74% (135 cases) and 19.00% (174 cases), respectively. Ocular and intestinal involvements overlapped in only 7 cases (0.76%). Male gender (74.8% vs. 51.1%, *P*=0.00), erythema nodosum (45.9% vs. 32.2%, *P*=0.01), and vascular involvement (6.7% vs. 1.7%, *P*=0.03) were more frequent in the ocular BS group compared with the intestinal BS group. On the contrary, hematologic involvement (7.5% vs. 0.0%, *P*=0.00) and fever (17.8% vs. 4.4%, *P*=0.00) were more frequent in the intestinal BS group compared with the ocular BS group. Additionally, the inflammation markers including ESR [26.5 (16.0–41.5) vs. 9.0 (5.0–15.0) mm/H, *P*=0.00], CRP [14.8 (4.8–33.0) vs. 4.1 (1.6–8.3) mg/L, *P*=0.00], serum amyloid A [27.4 (10.8–92.3) vs. 11.3 (6.0–24.0) mg/L, *P*=0.00], and interleukin 6 [8.4 (1.7–18.7) vs. 1.7 (1.5–3.2) pg/mL, *P*=0.00] were higher in the intestinal BS group than those in the ocular BS group, respectively.

**Conclusions:**

Ocular BS was more prevalent in male patients and more likely to manifest with erythema nodosum and vascular involvement, while intestinal BS tends to have fever and hematologic disorders with higher inflammation markers. Ocular BS and intestinal BS are two distinct clinical phenotypes and very rarely overlapped.

## Significance and innovations

Our study confirmed that ocular BS and intestinal BS were two distinct clinical phenotypes, and the eyes and intestines were rarely involved at the same time in patients with BS.

Ocular BS is more likely to manifest with erythema nodosum and vascular involvement, while intestinal BS tends to have fever and hematologic disorders with higher inflammation markers.

## Introduction

Behçet’s syndrome (BS) is a rare variant vasculitis which can involve the skin, mucosa, vessels, joints, nervous, eyes, and hematologic and gastrointestinal systems [1, 2]. BS is also known as the Silk Route disease with a high incidence in the Mediterranean, the Middle East, and the Far East, and the incidence rate in China is about 14/100,000 [1]. Since a triad of aphthous oral ulcers, genital lesions, and hypopyon was described by Benedict Adamantiades and Hulusi Behçet in the 1930s [3], neurological, cardiovascular, and intestinal systems involvements have also been reported [4–7]. Although BS is increasingly being recognized, investigated, and effectively managed, its pathogenesis remains unclear [1]. Additionally, the current diagnosis and efficacy evaluation of BS have mainly been dependent on clinical characteristics and there are no specific laboratory biomarkers for reference [8]. More and more evidence suggested BS tends to be a syndrome with several phenotypes rather than a disease [1, 2, 9–11]. In 2019, Seyahi reviewed and proposed six phenotypes: skin-mucosa involvement, joint involvement, vascular involvement, eye involvement, parenchymal neurological involvement, and gastrointestinal involvement [9]. Recently, our team divided BS patients in China into five clusters by cluster analysis: skin and mucosa type, joint involvement type, gastrointestinal type, uveitis type, and cardiovascular type [10]. Meanwhile, Soejima identified five independent clinical clusters among BS patients in Japan with a hierarchical cluster analysis, including a mucocutaneous subtype, a mucocutaneous with arthritis subtype, a gastrointestinal subtype, an eye subtype, and a neurological subtype [11]. Therefore, BS is a heterogeneous condition, with diverse clinical manifestations and prognoses [12].

Our previous cluster analysis results suggested BS patients in the uveitis type group rarely had intestinal involvement while no patients presented with ocular lesions in the gastrointestinal type [10], which were similar to those from the Japanese team [11]. The above evidences suggested that ocular BS and intestinal BS may be two phenotypes with different pathogenesis and clinical characteristics.

Nevertheless, there were rare studies on comparing ocular BS with intestinal BS. Most epidemiological studies mainly focused on a single subgroup of ocular BS or intestinal BS. Therefore, studies on the differences between ocular BS and intestinal BS could offer us more evidence to decipher the pathogenesis and hence provide precision medicine. In this study, we focused on the clinical characteristics and laboratory parameters between ocular BS and intestinal BS patients in China, aiming to find the differences between two phenotypes and help doctors and patients to better understand the clinical heterogeneity of BS.

## Patients and methods

### Patients

We conducted a retrospective analysis of BS patients who hospitalized in the Department of Rheumatology and Immunology of Huadong Hospital affiliated to Fudan University, from June 1, 2016, to June 1, 2021. This cross-sectional study was performed based on well-organized electronic medical records. All the above patients were from Shanghai Behçet’s Syndrome Database which was founded in 2012. Informed consent was signed by all patients before their personal information and data were included in Shanghai Behçet’s Syndrome Database. Patients’ records/information were anonymized and de-identified before analysis. The patients included in this study underwent an endoscopy, ocular examination, and imaging examination of blood vessels and the central nervous system (CNS) as part of their routine checkup. Organ involvement was assessed by reviewing the patient’s symptoms, past medical history, physical examination, laboratory studies, imaging examinations, and endoscopy findings, as previously reported [10]. When BS patients have uveitis and/or retinitis, they were diagnosed as ocular BS [13, 14]. The diagnosis of intestinal BS was made in accordance with previously established criteria based on colonoscopic features and clinical manifestations using a modified Delphi process [15, 16]. This study was approved by the medical ethics committee of Huadong Hospital affiliated to Fudan University with the following reference numbers: 2016K044 and 2018K031.

Inclusion criteria were as follows: (1) patients met the new International Criteria for Behçet’s Disease (ICBD) [17], and the final diagnosis was verified by at least 2 rheumatologists; (2) patients had complete clinical data and laboratory parameters; (3) patients had clinical symptoms related to BS in the past 1 month. And exclusion criteria were as follows: (1) the patients who were younger than 16 years old; (2) the patients having other disturbing diseases, such as infective diseases, other autoimmune diseases, endocrinal disorders, or malignancies (except myelodysplastic syndrome (MDS)); and (3) the patients who were pregnant or lactating.

### Clinical assessment

**Fig. 1:**
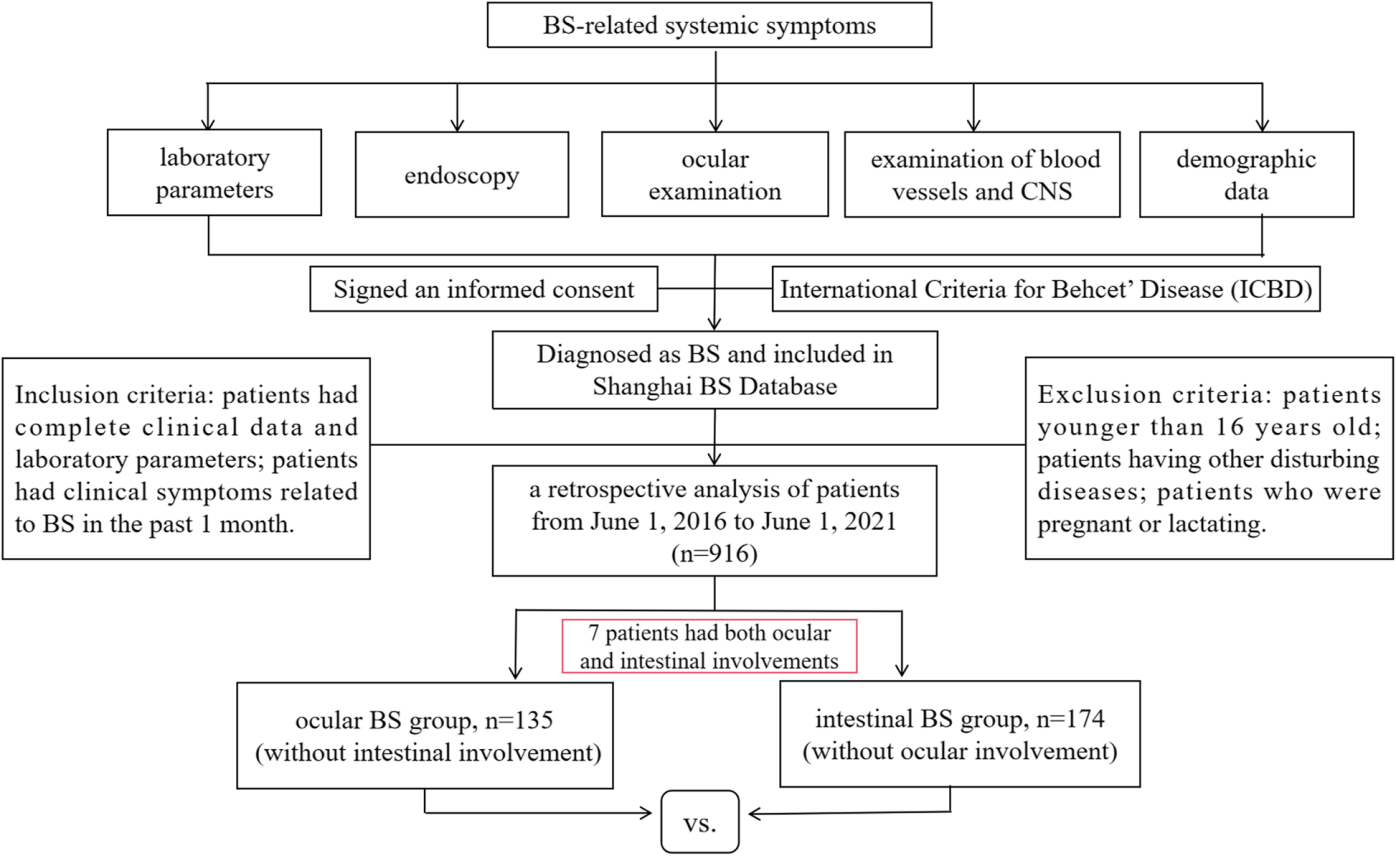
The flowchart of patients selection

The clinical manifestations during the course of disease and hospitalization in BS patients with ocular involvement and intestinal involvement were retrospectively extracted from the medical records. In this study, BS patients who had ocular without intestinal involvement were included in the ocular BS group, while BS patients who had intestinal without ocular involvement were included in the intestinal BS group. The flowchart of the patient selection is shown in Fig. 1. The disease activity scores of BS patients were recorded using the simplified Behçet’s Disease Current Activity Form (BDCAF) [18]. Patients were interviewed regarding their response to 12 clinical categories over the 4 weeks prior to the day of study enrollment, which composed the frame of BDCAF, and they were then scored from 0 to 12, but only with investigators’ agreements that symptoms were due to BS. Clinical categories were as follows: headache, mouth ulceration, genital ulceration, erythema nodosum, skin pustules, arthralgia, arthritis, nausea or vomiting or abdominal pain, diarrhea or bloody stool, ocular involvement, nervous system involvement, and major vessel involvement.

### Data collection

The following information was collected: gender, age at hospitalization, age at onset of first symptom, age at diagnosis of BS, period from oral ulcers to ocular or intestinal involvement, disease duration, history of hypertension, drinking history, smoking history, body mass index (BMI), the score of BDCAF, clinical manifestations of BS, clinical features of ocular BS patients (uveitis laterality, anatomic diagnosis, log of the minimum angle of resolution visual acuity (logMAR VA), ocular complications), the shape, number, diameter, location of intestinal ulcer, cytogenetic aberration of trisomy 8, tuberculosis (TB) infection T cell spot test (T-SPOT), pathergy test, fever, laboratory parameters (neutrophil-to-lymphocyte ratio (NLR), leukocyte, erythrocyte, hemoglobin, platelet, erythrocyte sedimentation rate (ESR), C-reactive protein (CRP), serum amyloid A (SAA), interleukin 6 (IL-6), immunoglobulin (IgA, IgG, IgE, IgM), complement 3 (C3), complement 4 (C4), CH50, and fecal occult blood test (FTOB)), and treatment approaches. The data of all included patients were verified by two investigators.

### Statistical analysis

The software of SPSS version 23.0 (SPSS Inc., Chicago, IL, USA) was used for statistical analysis. Data were assessed for normality of distribution using the Shapiro-Wilk test first. Categorical variables were denoted by percentage or ratio. Qualitative variables were expressed as median (25–75% interquartile range [IQR]).

The Mann-Whitney *U* test and Pearson chi-square or continuity correction were used for quantitative and categorical variables, respectively. Differences were considered statistically significant when *P* was less than 0.05 (*P* < 0.05).

## Results

### Demographic and clinical manifestations of ocular BS and intestinal BS patients

A total of 916 BS patients were enrolled in this study. Of the cohort of 916 BS patients, 135 (14.74%) patients who had ocular involvement without intestinal involvement were included in the ocular BS group, while 174 (19.00%) patients who had intestinal involvement without ocular involvement were included in the intestinal BS group. The sex ratio (male:female) in the ocular BS group was 2.97:1, while in the intestinal BS group was 1.05:1 (*P*=0.00). The median age at hospitalization (IQR) in the ocular BS group was 35.0 (27.0–44.0) years while in the intestinal BS group was 33.5 (24.0–45.0) years (*P*=0.52). The median age at onset of oral ulcers (IQR) in the ocular BS group was 26.0 (20.0–34.0) years while in the intestinal BS group was 25.0 (15.0–36.0) years (*P*=0.16). The median age at diagnosis of BS (IQR) in the ocular BS group was 32.0 (26.0–40.0) years while in the intestinal BS group was 31.5 (22.8–43.3) years (*P*=0.76). The median period from oral ulcers to ocular involvement (IQR) in the ocular BS group was 3.5 (1.0–7.0) years while the median period from oral ulcers to intestinal involvement (IQR) in the intestinal BS group was 5.0 (2.0–9.1) years (*P*=0.02). The median disease duration (IQR) in the ocular BS group was 7.0 (4.0–10.0) years while in the intestinal BS group was 6.0 (2.0–10.0) years (*P*=0.43). The median BMI (IQR) in the ocular BS group was 23.1 (21.0–25.2) years while in the intestinal BS group was 21.2 (18.7–23.7) years (*P*=0.00). The most common clinical manifestations during the course of disease in ocular BS patients were oral ulcers (100.0%) and ocular involvement (100.0%), followed by genital ulcers (71.1%) and erythema nodosum (45.9%), pseudofolliculitis (41.5%), arthritis or arthralgia (11.1%), vascular involvement (6.7%), fever (4.4%), and nervous system involvement (3.0%). The most common clinical manifestations during the course of disease in intestinal BS patients were oral ulcers (100.0%) and gastrointestinal lesions (100.0%), followed by genital ulcers (72.4%) and erythema nodosum (32.3%), pseudofolliculitis (31.0%), fever (17.8%), arthritis or arthralgia (17.2%), hematologic involvement (7.5%), vascular involvement (1.7%), and nervous system involvement (1.2%). The detailed demographic and clinical manifestations of the above two groups are shown in Table 1. There was a statistically significant difference in terms of gender, period from oral ulcers to ocular or intestinal involvement, BMI, fever, erythema nodosum, vascular involvement, and hematologic involvement between the ocular and the intestinal BS groups (all *P*<0.05). Male gender (74.8% vs. 51.1%, *P*=0.00), erythema nodosum (45.9% vs. 32.2%, *P=*0.01), and vascular involvement (6.7% vs. 1.7%, *P*=0.03) were more frequent in ocular BS compared with the intestinal BS group. Hematologic involvement (7.5% vs. 0.0%, *P*=0.00) and fever (17.8% vs. 4.4%, *P*=0.00) were more frequent in intestinal BS compared with the ocular BS group. While there was no statistically significant difference between the two groups in terms of age at hospitalization, at onset of oral ulcers, and at diagnosis of BS, disease duration, history of hypertension, drinking history, smoking history, BDCAF, T-SPOT (+), pathergy test (+), oral ulcers, genital ulcers, pseudofolliculitis, arthritis or arthralgia, and nervous system involvement between two groups.

**Table 1:** Demographic and clinical manifestations of ocular BS and intestinal BS groups

Variables	Ocular BS (*n*=135)	Intestinal BS (*n*=174)	*P*
Gender (male), *n* (%)	101 (74.8%)	89 (51.1%)	0.00*
Age at hospitalization (IQR), (years)	35.0 (27.0–44.0)	33.5 (24.0–45.0)	0.52
Age at onset of oral ulcers (IQR), (years)	26.0 (20.0–34.0)	25.0 (15.0–36.0)	0.16
Age at diagnosis of BS (IQR), (years)	32.0 (26.0–40.0)	31.5 (22.8–43.3)	0.76
Period from oral ulcers to ocular/intestinal involvement (IQR), (years)	3.5 (1.0–7.0)	5.0 (2.0–9.1)	0.02*
Disease duration (IQR), (years)	7.0 (4.0–10.0)	6.0 (2.0–10.0)	0.43
BMI (IQR)	23.1 (21.0–25.2)	21.2 (18.7–23.7)	0.00*
History of hypertension (yes), *n* (%)	2.0 (1.5%)	3.0 (1.7%)	1.00
Drinking history (yes), *n* (%)	3.0 (2.2%)	0.0 (0.0%)	0.16
Smoking history (yes), *n* (%)	6 (4.4%)	3 (1.7%)	0.29
BDCAF (IQR)	1 (1–2)	1 (1–2)	0.66
T-SPOT (+), *n* (%)	20 (14.8%)	38 (21.8%)	0.12
Pathergy test (+), *n* (%)	43 (31.9%)	62 (35.6%)	0.49
Fever, *n* (%)	6 (4.4%)	31 (17.8%)	0.00*
Oral ulcers, *n* (%)	135 (100.0%)	174 (100.0%)	1.00
Genital ulcers, *n* (%)	96 (71.1%)	126 (72.4%)	0.80
Erythema nodosum, *n* (%)	62 (45.9%)	56 (32.2%)	0.01*
Pseudofolliculitis, *n* (%)	56 (41.5%)	54 (31.0%)	0.06
Arthritis or arthralgia, *n* (%)	15 (11.1%)	30 (17.2%)	0.13
Vascular involvement, *n* (%)	9 (6.7%)	3 (1.7%)	0.03*
Nervous system involvement, *n* (%)	4 (3.0%)	2 (1.2%)	0.47
Ocular involvement, *n* (%)	135 (100.0%)	0 (0.0%)	0.00*
Gastrointestinal lesions, *n* (%)	0 (0.0%)	174 (100.0%)	0.00*
Hematologic involvement, *n* (%)	0 (0.0%)	13 (7.5%)	0.00*

*BMI* body mass index, *BDCAF* Behcet’s Disease Current Activity Form, *T-SPOT* tuberculosis (TB) infection T cell spot test. *P<0.05

Furthermore, only 7 (0.76%) patients in the cohort of 916 BS patients had both ocular and intestinal involvements. The 7 patients consisted of 5 males and 2 females. The median age at hospitalization (IQR) of the 7 patients was 29.0 (23.0–46.0) years. The median age at onset of oral ulcers (IQR) and age at diagnosis of BS (IQR) was 18.0 (13.0–27.0) and 28.0 (22.0–44.0) years. The median period from oral ulcers to ocular involvement (IQR) and the period to intestinal involvement (IQR) was 7.0 (1.0–19.0) and 2.0 (0.5–20.0) years. The median disease duration (IQR) of the 7 patients was 9.0 (2.0–20.0) years. The percentage of T-SPOT (+) and pathergy test (+) in the 7 patients was 42.9% and 14.3%, respectively. The most common clinical manifestations during the course of disease in the 7 patients were oral ulcers (100.0%), ocular involvement (100.0%), and gastrointestinal lesions (100.0%), followed by arthritis or arthralgia (42.9%), fever (28.6%), pseudofolliculitis (28.6%), and genital ulcers (14.3%).

### Laboratory parameters of ocular BS and intestinal BS patients

The laboratory parameters between ocular BS and intestinal BS groups were compared in this study. There was a statistically significant difference in terms of ESR, CRP, erythrocyte, leukocyte, hemoglobin, IgG, SAA, IL-6, C3, C4, and CH50 between the two groups (all *P*<0.05). Inflammation markers including ESR, CRP, SAA, and IL-6 in the intestinal BS group seemed to be higher than those in the ocular BS group. On the other hand, erythrocyte and hemoglobin were lower in the intestinal BS group than those in the ocular BS group. While there was no statistically significant difference between the two groups in terms of platelets, NLR, IgA, IgE, and IgM (*P*>0.05). The detailed laboratory parameters of the above two groups are shown in Table 2.

**Table 2:** Laboratory parameters of ocular BS and intestinal BS groups

Variables	Ocular BS (*n*=135)	Intestinal BS (*n*=174)	*P*
Erythrocyte (IQR), (10^9^/L)	4.7 (4.3–5.0)	4.3 (3.9–4.7)	0.00*
Leukocyte (IQR), (10^9^/L)	8.0 (6.1–10.6)	6.5 (5.2–9.2)	0.00*
NLR (IQR),	2.55 (1.74–4.67)	2.3 (1.7–3.7)	0.31
Hemoglobin (IQR), (g/L)	141 (132–150)	124 (108–136)	0.00*
Platelets (IQR), (10^9^/L)	224 (183–275)	124 (191–298)	0.09
ESR (IQR), (mm/H)	9.0 (5.0–15.0)	26.5 (16.0–41.5)	0.00*
CRP (IQR), (mg/L)	4.1 (1.6–8.3)	14.8 (4.8–33.0)	0.00*
SAA (IQR), (mg/L)	11.3 (6.0–24.0)	27.4 (10.8–92.3)	0.00*
IL-6 (IQR), (pg/mL)	1.7 (1.5–3.2)	8.4 (1.7–18.7)	0.00*
IgA (IQR), (g/L)	2.30 (1.67–3.13)	2.45 (1.85–3.33)	0.16
IgG (IQR), (g/L)	9.78 (8.13–11.71)	11.13 (9.38–13.62)	0.00*
IgM (IQR), (g/L)	1.04 (0.76–1.53)	1.12 (0.85–1.50)	0.29
IgE (IQR), (IU/ML)	20 (10–73)	33 (10–76)	0.07
C3 (IQR), (g/L)	1.20 (1.06–1.32)	1.26 (1.12–1.41)	0.01*
C4 (IQR), (g/L)	0.22 (0.17–0.29)	0.25 (0.20–0.31)	0.00*
CH50 (IQR), (g/L)	49 (45–55)	54 (47–61)	0.00*

*NLR* neutrophil-to-lymphocyte ratio, *ESR* erythrocyte sedimentation rate, *CRP* C-reactive protein, *SAA* serum amyloid A, *IL-6* interleukin 6, *C3* complement 3, *C4* complement 4. *P<0.05

### Clinical features of the ocular BS group

Of the cohort of 135 ocular BS patients, 102 (75.5%) patients had bilateral uveitis and 33 (24.5%) patients had unilateral uveitis. Of the unilateral cases, 17 (12.6%) cases were right eye involvements and 16 (11.9%) were left eye involvements. Of 237 involved eyes, the predominant types of ocular inflammation were posterior uveitis, panuveitis, and anterior uveitis, occurring in 41.8%, 36.3%, and 21.9%, respectively. Additionally, of 237 involved eyes, vitritis was the most commonly observed sign in 185 eyes (78.1%). Retinal vasculitis was observed in 102 eyes (43.0%). The median logMAR VA (IQR) of the right eye was 0.3 (1–0.05) and the left eye was 0.3 (1.3–0). Of 237 involved eyes, the percentage of eyes with no light perception, hand motion, and light perception was 5.9%, 3.5%, and 7.2%, respectively. In addition, cataract was observed in 18 eyes (7.6%) while glaucoma was observed in 3 eyes (1.3%). All the detailed clinical features of the ocular BS group are shown in Table 3.

**Table 3:** Clinical features of the ocular BS group (*n*=135)

Variables	*N* (%) or median (IQR)
Uveitis laterality (*n*=135)	
Right eye	17 (12.6%)
Left eye	16 (11.9%)
Bilateral	102 (75.5%)
Anatomic diagnosis (*n*=237)	
Anterior uveitis	52 (21.9%)
Posterior uveitis	99 (41.8%)
Panuveitis	86 (36.3%)
Vitritis	185 (78.1%)
Retinal vasculitis	102 (43.0%)
LogMAR VA	
OD, median (IQR)	0.3 (1–0.05)
OS, median (IQR)	0.3 (1.3–0)
No light perception, *n* (%)	14 (5.9%)
Hand motion, *n* (%)	13 (5.5%)
Light perception, *n* (%)	17 (7.2%)
Ocular complications	
Cataract, *n* (%)	18 (7.6%)
Glaucoma, *n* (%)	3 (1.3%)

*logMAR VA* log of the minimum angle of resolution visual acuity, *OD* oculus dexter, *OS* oculus sinister

Of the 7 BS patients who had both ocular and intestinal involvements, 6 (85.7%) patients had bilateral uveitis and 1 (14.3%) patient had unilateral uveitis. The percentage of eyes with posterior uveitis, panuveitis, and anterior uveitis was 76.9%, 7.7%, and 15.4%, respectively. Moreover, the median logMAR VA (IQR) of the right eye was 0 (0.1–0) and the left eye was 0.1 (1–0). Uveitis in 71.4% patients were past lesions without inflammatory activity.

### Clinical features of the intestinal BS group

Of the cohort of 174 intestinal BS patients, 25 (14.4%) patients underwent the surgical treatment due to ulcer perforation. Nausea or vomiting or abdominal pain or diarrhea or hematochezia only happened in 69 (39.7%) patients while 105 (60.3%) patients had no gastrointestinal symptoms. FTOB (+) was observed in only 44 (25.3%) patients. Of the 13 patients with hematologic involvement in the intestinal BS group, 10 (76.9%) patients had cytogenetic aberration of trisomy 8 and diagnosed as MDS. The most common intestinal segment involved in the intestinal BS group was terminal ileum (50.6%), followed by ileocecal valve (36.2%), colon (24.7%), small intestine (10.3%), cecum (3.4%), rectum (3.4%), and anastomotic ulcer of ileocecal area (1.7%), while the least common was duodenum (1.1%) and perianal (1.1%). The most common shape of intestinal ulcer was round/annular/oval shape (79.3%), then irregular shape (18.4%) and longitudinal shape (2.3%). The diameter of intestinal ulcers ≥ 2cm was observed in 38 (21.8%) patients while < 2cm was observed in 136 (78.2%) patients. Additionally, 60 (34.5%) patients had a solitary single intestinal ulcer, while 114 (65.5%) had more than one intestinal ulcers. The most common location of the solitary ulcer was ileocecal valve (51.7%), followed by terminal ileum (40.0%), anastomotic ulcer of ileocecal area (5.0%), and colon (3.3%). All the detailed clinical features of intestinal BS group are shown in Table 4.

**Table 4:** Clinical features of the intestinal BS group (*n*=174)

Variables	*N* (%)
Ulcerative shape	
Irregular shape	32 (18.4%)
Round/annular/oval shape	138 (79.3%)
Longitudinal shape	4 (2.3%)
Number of ulcers	
*N*=1	60 (34.5%)
*N*>1	114 (65.5%)
Diameter of ulcers	
≥2cm	38 (21.8%)
<2cm	136 (78.2%)
Location of ulcers	
Esophagus	11 (6.3%)
Gastric antrum	3 (1.7%)
Duodenum	2 (1.1%)
Small intestine	18 (10.3%)
Terminal ileum	88 (50.6%)
Ileocecal valve	63 (36.2%)
Cecum	6 (3.4%)
Colon	43 (24.7%)
Rectum	6 (3.4%)
Perianal	2 (1.1%)
Anastomotic ulcer of ileocecal area	3 (1.7%)
Surgical treatment due to ulcer perforation	25 (14.4%)
Nausea or vomiting or abdominal pain or diarrhea or hematochezia	69 (39.7%)
FTOB (+)	44 (25.3%)
Location of solitary single ulcer (*n*=60)	
Ileocecal valve	31 (51.7%)
Terminal ileum	24 (40.0%)
Anastomotic ulcer of ileocecal area	3 (5.0%)
Colon	2 (3.3%)
Features with hematologic involvement (*n*=13)	
Cytogenetic aberration of trisomy 8	10 (76.9%)
Diagnosed as MDS	10 (76.9%)

*FTOB* fecal occult blood test, *MDS* myelodysplastic syndrome

Of the 7 BS patients who had both ocular and intestinal involvements, no patient had hematologic involvement nor underwent the surgical treatment due to ulcer perforation. Five (71.4%) patients had no gastrointestinal symptoms. FTOB (+) was observed in only 2 (28.6%) patients. The most common intestinal segment involved in the intestinal BS group was ileocecal valve (42.9%), followed by terminal ileum (28.6%), colon (28.6%), and cecum (14.3%). The most common shape of intestinal ulcer was round/annular/oval shape (100%). The diameter of intestinal ulcers ≥ 2cm was observed in 1 (14.3%) patient while < 2cm was observed in 6 (85.7%) patients. Additionally, 3 (42.9%) patients had a solitary single intestinal ulcer, while 4 (57.1%) had more than one intestinal ulcers. The most common location of the solitary ulcer was ileocecal valve (14.3%), terminal ileum (14.3%), and colon (14.3%).

### Treatment approaches

In this study, glucocorticoid, thalidomide, and cyclosporine were the most commonly used drugs in the ocular BS and intestinal BS patients. However, sulfasalazine and mesalazine were more used in the intestinal BS patients. Furthermore, biologics, colchicine, tofacitinib, hydroxychloroquine, cyclophosphamide, and methotrexate were also used in the two groups. One hundred nine (62.6%) patients used biologics in the intestinal BS group while only 44 (32.6%) patients used biologics in the ocular BS group. However, in the ocular BS group, more patients used tofacitinib than that in the intestinal BS group. The information of treatment approaches between ocular and intestinal BS groups is shown in Table 5.

**Table 5:** Therapeutic drugs for ocular BS and intestinal BS patients

Overall treatment	Ocular BS (*n*=135, %)	Intestinal BS (*n*=174, %)
Glucocorticoid	116 (85.9)	167 (96.0)
Sulfasalazine	0 (0.0)	104 (59.8)
Mesalazine	0 (0.0)	15 (8.6)
Colchicine	37 (27.4)	40 (23.0)
Thalidomide	128 (94.8)	150 (86.2)
Hydroxychloroquine	12 (8.9)	17 (9.8)
Cyclosporine	98 (72.6)	147 (84.5)
Tofacitinib	27 (20.0)	6 (3.4)
Cyclophosphamide	4 (3.0)	2 (1.1)
Methotrexate	2 (1.5)	1 (0.6)
Biologics	44 (32.6)	109 (62.6)

## Discussion

BS is a heterogeneous condition, with diverse clinical manifestations and prognoses [12]. More and more investigators refer to BS as “Behçet’s syndrome” [1, 2]. As known to all, ocular and intestinal involvements were the common major organ involvements in BS. Recently, some researchers identified some independent clinical clusters including both a gastrointestinal subtype and an eye subtype [9–11]. However, there were rarely studies comparing the above two subtypes. Herein, we focused on the clinical characteristics and laboratory parameters between ocular BS and intestinal BS patients in China, aiming to find the differences between two phenotypes and help doctors and patients to better understand the heterogeneity of BS.

In the cohort of 916 BS patients, 135 (14.74%) patients who had ocular involvement without intestinal involvement were included in the ocular BS group, while 174 (19.00%) patients who had intestinal involvement without ocular involvement were included in the intestinal BS group. Only 7 (0.76%) patients in the cohort had both ocular and intestinal involvement which suggested ocular BS and intestinal BS may be two phenotypes with different pathogenesis and clinical characteristics. The frequency of intestinal involvement in our cohort was 19.00% which was in line with those from previous reports in China and Japan [10, 11, 19], but higher than that from Turkey and Iran [20, 21]. The frequency of ocular involvement in our cohort was lower than that reported before [11, 20, 22], which may be associated with that the patients with ocular symptoms firstly go to the ophthalmology clinic. Previous studies have reported that the prevalence rate of uveitis in BS patients is higher in males and serious ocular disease is also more frequent in males as compared with females [10, 11, 20–23]. In our study, male were also found more likely to have ocular involvement in ocular BS, while the ratio of male to female in intestinal BS was almost similar. Moreover, we found ocular manifestations usually occur 3.5 (IQR, 1.0–7.0) years after the onset of oral ulcers, while intestinal manifestations occur 5.0 (IQR, 2.0–9.1) years after the onset of oral ulcers. This may suggest ocular manifestations appear earlier than intestinal manifestations. In this cohort, erythema nodosum and vascular involvement were more commonly observed in ocular BS, while fever and hematologic involvement were more commonly observed in intestinal BS. Shen reported that MDS was more commonly observed in intestinal BS patients [24], which was similar to our finding. Furthermore, fever has been reported common in intestinal BS patients [20, 25]. The frequency of pathergy test (+) was about 30% in two groups, which was lower than 50.4% reported in Iran [21]. And there was no statistically significant difference between two groups in terms of the pathergy test (+) in our cohort.

By comparing the laboratory parameters between ocular BS and intestinal BS groups, we found that some inflammation markers including ESR, CRP, SAA, and IL-6 in the intestinal BS group were higher than those in the ocular BS group. There were few studies on the difference in laboratory parameters between BS patients with ocular involvement and intestinal involvement. Balbaba reported ESR and CRP were significantly higher in ocular active BS patients compared with ocular inactive BS patients and healthy control subjects [26]. However, Cai found no significant differences in terms of ESR and CRP between uveitis recurrence BS patients and non-uveitis recurrence patients [27]. SAA might be potential predictive factors for uveitis recurrence in BS patients [27]. In addition, SAA in intestinal BS patients was found significantly higher than that in controls [28]; the levels of SAA showed a better correlation with disease activity than CRP [29]. Our previous study also found ESR (≥15mm/H), CRP (>10mg/L), and IL-6 (>7pg/mL) were the independent risk factors of intestinal involvement in BS patients [19]. This study was the first to report that BS patients with intestinal involvement were more likely to have elevated inflammation markers than those with ocular involvement, which suggested ocular BS and intestinal BS may be two phenotypes with different pathogenesis.

On the other hand, erythrocyte and hemoglobin were lower in the intestinal BS group than those in the ocular BS group. Lower hemoglobin in the intestinal BS patients has been reported by some studies [19, 30]. This may be related to the poor absorption function of BS patients with intestinal involvement, which is more prone to anemia and poor nutritional levels.

Ocular inflammation in BS can involve all the uveal tract; for this reason, uveitis can be anterior (11%), posterior (28.8%), or panuveitis (60.2%) [31]. In our cohort, most ocular BS patients had bilateral uveitis, which was consistent with the previous studies [31, 32]. However, the predominant types of ocular inflammation were posterior uveitis (41.8%), panuveitis (36.3%), and anterior uveitis (21.9%), respectively. About 16.6% of affected eyes have severe visual impairment including no light perception, hand motion, and light perception. A study from two referral centers in England and in Australia found that male sex, unilateral disease, and left eye involvement increased the risks of severe visual loss at 5 and 10 years [33]. Ocular complications in ocular BS are diverse and include commonly cataract (15–77%), ocular hypertension (14–31%) or glaucoma (19%), macular edema (25–44%), epiretinal membrane (10–17%) and optic atrophy (8–24%), and retinal detachment (1.4–11%) [31, 34, 35]. In our study, cataract was observed in 18 eyes (7.6%) while glaucoma was observed in 3 eyes (1.3%). This could be due to the late initial presentation to the ophthalmologist, recurrent inflammation, and long-term use of corticosteroids.

The intestinal involvement is much more frequent in patients in East Asian countries including China and Japan than in any regions [10, 11, 19]. In our cohort, 19.00% patients who had intestinal involvement without ocular involvement were included in the intestinal BS group. The most common intestinal segment involved in the intestinal BS group was the terminal ileum, which was consistent with previous studies [19, 36]. Additionally, we found 105 (60.3%) intestinal BS patients having typical ulcers under colonoscopy without gastrointestinal symptoms. Therefore, it is necessary for BS patients in China to undergo colonoscopy. Intestinal ulceration is a common clinical feature in BS associated with bone marrow failure, classified as conditions such as MDS, and associated with trisomy 8 [24, 37, 38]. In line with those reports, we found 10 patients had cytogenetic aberration of trisomy 8 and diagnosed as MDS in the 13 patients with hematologic involvement of the intestinal BS group. Therefore, it is recommended for intestinal BS patients with hematologic involvement to undergo chromosomal evaluation.

Several limitations should be noted. Firstly, our results are not necessarily being generalizable to other countries because most of the patients were Chinese who had relatively unique and homogenous genetic backgrounds. Secondly, selection bias was unavoidable because of the study design. Finally, our study was conducted in a single center. Therefore, multi-center samples are needed for verification of the differences between ocular BS and intestinal BS groups in the future.

## Conclusion

This study compared the clinical characteristics and laboratory parameters in ocular BS and intestinal BS patients in China. We confirmed that ocular BS was more prevalent in male patients and more likely to have erythema nodosum and vascular involvement, while intestinal BS tends to have fever and hematologic involvement accompanied with elevated inflammation markers including ESR, CRP, SAA, and IL-6. Ocular BS and intestinal BS are two distinct clinical phenotypes that rarely overlapped. Hence, higher attention shall be paid to these differences between ocular BS and intestinal BS.

## Data Availability

The datasets used and analyzed during the current study are available from the corresponding author on reasonable request.

## Abbreviations

BS: Behçet’s syndrome
BDCAF: Behçet’s Disease Current Activity Form
NLR: Neutrophil-to-lymphocyte ratio
SAA: Serum amyloid A
CRP: C-reactive protein
ESR: Erythrocyte sedimentation rate
IL-6: Interleukin 6
C3: Complement 3
C4: Complement 4
BMI: Body mass index
T-SPOT: Tuberculosis (TB) infection T cell spot test
logMAR VA: Log of the minimum angle of resolution visual acuity
OD: Oculus dexter
OS: Oculus sinister
FTOB: Fecal occult blood test
MDS: Myelodysplastic syndrome
